# Formulation of a Three-Component Essential Oil Mixture from *Lavandula dentata*, *Rosmarinus officinalis*, and *Myrtus communis* for Improved Antioxidant Activity

**DOI:** 10.3390/ph17081071

**Published:** 2024-08-15

**Authors:** Amine Elbouzidi, Mohamed Taibi, Naoufal El Hachlafi, Mounir Haddou, Mohamed Jeddi, Abdellah Baraich, Aya Aouraghe, Reda Bellaouchi, Ramzi A. Mothana, Mohammed F. Hawwal, François Mesnard, Christophe Hano, Abdeslam Asehraou, Khalid Chaabane, Bouchra El Guerrouj, Mohamed Addi

**Affiliations:** 1 Laboratoire d’Amélioration des Productions Agricoles, Biotechnologie et Environnement (LAPABE), Faculté des Sciences, Université Mohammed Premier, Oujda 60000, Morocco; mohamedtaibi9@hotmail.fr (M.T.); haddou.mounir27@gmail.com (M.H.); k.chaabane@ump.ac.ma (K.C.); elguerroujb@gmail.com (B.E.G.); 2 Centre de l’Oriental des Sciences et Technologies de l’Eau et de l’Environnement (COSTEE), Université Mohammed Premier, Oujda 60000, Morocco; aya.aouraghe@ump.ac.ma; 3 Laboratory of Microbial Biotechnology and Bioactive Molecules, Faculty of Sciences and Technologies, Sidi Mohamed Ben Abdellah University, Imouzzer Road, Fez P.O. Box 2202, Morocco; naoufal.elhachlafi@usmba.ac.ma (N.E.H.); mohamed.jeddi@usmba.ac.ma (M.J.); 4 Laboratory of Bioresources, Biotechnology, Ethnopharmacology and Health, Faculty of Sciences, Mohammed First University, Boulevard Mohamed VI, B.P. 717, Oujda 60000, Morocco; abdellah.baraich@ump.ac.ma (A.B.); r.bellaouchi@ump.ac.ma (R.B.); asehraou@yahoo.fr (A.A.); 5 Department of Pharmacognosy, College of Pharmacy, King Saud University, Riyadh 11451, Saudi Arabia; rmothana@ksu.edu.sa (R.A.M.); mhawwal@ksu.edu.sa (M.F.H.); 6 UMRT INRAE 1158 BioEcoAgro, Laboratoire BIOPI, University of Picardie Jules Verne, 80000 Amiens, France; francois.mesnard@u-picardie.fr; 7 Institut de Chimie Organique et Analytique, Université d’Orléans-CNRS, UMR 7311 BP 6759, CEDEX 2, 45067 Orléans, France; hano.christophe@gmail.com

**Keywords:** *Lavandula dentata*, *Rosmarinus officinalis*, *Myrtus communis*, essential oils, experimental mixture design, antioxidant activity, food preservation, biopharmaceuticals

## Abstract

The optimization of existing natural antioxidants that are highly effective is crucial for advancements in medicine and the food industry. Due to growing concerns regarding the safety of synthetic antioxidants, researchers are increasingly focusing on natural sources, particularly essential oils (EOs). Combining EOs might enhance antioxidant activity due to increased chemical diversity. This study investigates, for the first time, the antioxidant properties of EOs from *Lavandula dentata*, *Rosmarinus officinalis*, and *Myrtus communis*, both individually and in combination, using the augmented-simplex design methodology. The in vitro evaluation of the antioxidant activity was performed using DPPH and ABTS radical scavenging assays. Chromatography gas-mass spectrometry (CG-MS) revealed that 1,8-cineol (37.27%) and pinocarveol (12.67%) are the primary components of *L. dentata*; verbenone (16.90%), camphor (15.00%), and camphene (11.03%) are predominant in *R. officinalis*; while cineol (43.32%) is the main component of M. communis. The EOs showed varying scavenging activities against ABTS and DPPH radicals, with DPPH assay values ranging from 194.10 ± 3.01 to 541.19 ± 3.72 µg/mL and ABTS assay values ranging from 134.07 ± 1.70 to 663.42 ± 2.99 µg/mL. These activities were enhanced when the EOs were combined. The optimal antioxidant blend for DPPH_IC50_ consisted of 20% *L. dentata*, 50% *R. officinalis*, and 30% *M. communis*. For the highest ABTS radical scavenging activity, the best combination was 18% *L. dentata*, 43% *R. officinalis*, and 40% *M. communis*. These results highlight the potential of EO combinations as new natural formulations for use in cosmeceutical, food, and pharmaceutical sectors.

## 1. Introduction

The search for novel, safe, and efficient antioxidants derived from natural sources to prevent reactive chemical species (RCS)-induced oxidative damage to live cells has garnered a lot of attention lately [1]. Because they have an unpaired electron in its valence shell, free radicals—the main RCS involved in oxidation—are extremely reactive. Important free radicals that can change essential macromolecules, including lipids, DNA, and proteins, include hydroxyl radicals (HO·), hydrogen peroxide (H_2_O_2_), superoxide anions (O_2_^−^), singlet oxygen (1O_2_), peroxynitrite (NO_3_^−^), and nitric oxide (·NO) [2]. This change results in cellular damage and upsets normal homeostasis, which can lead to a number of pathological disorders, including infections, cancer, inflammatory illnesses, and cardiovascular diseases [3,4,5,6]. In order to protect physiological processes, external antioxidants can assist in sustaining the equilibrium between free radicals and the antioxidant system. Butylated hydroxyanisole (BHA) and butylated hydroxytoluene (BHT), two commercial antioxidants, have recently been found to pose health risks [7]. Similar to this, there may be negative health effects from other synthetic antioxidants such propyl gallate (PG) and tert-butylhydroquinone (TBHQ). As a result, scientists are creating novel, safe antioxidant compositions using natural materials, especially essential oils (EOs) [7].

EOs, secondary metabolites derived from plants, are known for their antioxidant properties [8]. The direct antioxidant activity of EOs, through free radical scavenging and inhibition of linoleic acid oxidation, can benefit the food industry by enhancing the storage stability of food products [9,10]. However, EO aromas should not alter the sensory properties of food. Research on the antioxidant activity of EOs in real systems has shown that whole EOs, containing multiple active compounds, or mixtures of isolated EO compounds can extend the shelf life of food products [11,12]. Additionally, EO vapors have been identified as components of active packaging [13], and incorporating antioxidant EOs directly into polymer films of packaging has been proposed to reduce microbial decay and maintain the antioxidant properties of food [14].

Toothed lavender (*Lavandula dentata* L.), belonging to the *Lamiaceae* family, is native to parts of the Mediterranean and can be identified by its distinctive serrated leaves and pale purple flowers. It is less common than its relative, *Lavandula angustifolia*, but is used similarly in decorative and culinary applications [15,16]. In traditional medicine, toothed lavender is primarily used for its calming effects. It is reputed to alleviate anxiety, promote relaxation, and aid in sleep, which makes it a popular choice in aromatherapy [17]. The essential oil of *L. dentata* is noted for its antioxidative, antifungal, and antiseptic properties [18]. Its major components include linalool, camphor, and cineole, which contribute to its overall therapeutic efficacy [17]. Rosemary (*Rosmarinus officinalis* L.), belongs to the *Lamiaceae* family, is an evergreen shrub native to the Mediterranean region. It features needle-like leaves and is adorned with blue flowers, making it not only culinary but also aesthetically appreciated [19]. Traditionally, rosemary has been cherished for enhancing memory and alleviating muscle pain and spasms. It has also been used for its digestive properties, particularly in Mediterranean folk medicine [20]. Rosemary exhibits a range of pharmacological activities including antioxidant, antimicrobial, and anti-inflammatory effects. These properties are largely attributed to its rich content of rosmarinic acid, carnosic acid, and essential oils like cineole and camphor [21]. 

Myrtle (*Myrtus communis* L.), belonging to the *Myrtaceae* family, is an evergreen shrub with glossy, aromatic leaves and star-shaped white flowers, followed by blue-black berries. It is indigenous to the Mediterranean and Western Asia and is often used in ornamental landscaping [22]. Historically, myrtle has been used to treat a variety of ailments, including respiratory and digestive issues. It has been used in herbal medicine as an antiseptic and astringent agent [23]. Myrtle is known for its antioxidant, anti-inflammatory, and antiseptic properties. These are largely due to its high phenolic content, including myrtenol, which is effective in treating chronic inflammation and preventing infection [24].

Recent studies have intriguingly shown that combining essential oils (EOs) can significantly enhance their antioxidant properties. Although the precise mechanisms and optimal proportions for these synergistic interactions are not yet fully understood, gaining such insights is crucial for identifying novel and effective EO combinations. To explore this, we devised a new approach to evaluate the antioxidant potential arising from the interactions of three specific oils: *Lavandula dentata*, *Rosmarinus officinalis*, and *Myrtus communis*. Utilizing the augmented-simplex design methodology, this study is the first of its kind on the three previously stated plants. This approach determines the ideal EO concentrations that produce a synergistic antioxidant effect for pharmaceutical and food preservation purposes.

## 2. Results

### 2.1. Chemical Profile of the Three EOs

Table 1 presents the chemical profiles, molecular formulas, percentages, and yields of essential oils (EOs) from *L. dentata*, *R. officinalis*, and *M. communis* (TIC chromatograms, and the composition along with retention times, are displayed in the Supplementary Materials Figures S1–S3 and Tables S1–S3). The yields of the EO extracts are 0.55, 1.33, and 0.31 (*v/w*), respectively. Each EO contains different numbers of phytoconstituents: seventeen for *L. dentata*, nineteen for *R. officinalis*, and fourteen for *M. communis*, accounting for 100%, 98.47%, and 100% of the total composition of their respective plants. In *L. dentata* EO, cineol is the predominant compound at 37.27%, followed by the oxygenated monoterpene pinocarveol at 12.67%. Previous studies, including those by Touati et al. [25] and Bousmaha et al. [26], confirm the predominance of 1,8-cineol in *L. dentata* EO, with percentages ranging from 0.9 to 36.3%, while another study by Msaada et al. [27], on Tunisian *L. dentata*, found linalool (47.30%) as the major compound in this EO.

For *R. officinalis*, of the nineteen detected compounds, 60.50% are oxygenated monoterpenes, with verbenone leading at 16.9%, camphor with 15%, and camphene with 11.03%. Other minor compounds include *p*-linalool at 6.86%, a terpenic alcohol known for its sedative and anxiolytic effects, α-pinene at 6.1%, and cineol at 4.97%. These findings align with the study by Anwar et al., highlighting linalool as a principal component at 29.1%, followed by 1,8-cineole at 18.4%. 

For *M. communis* EO, the major compounds include cineole (43.32%), a compound known for its expectorant, anti-inflammatory, and antimicrobial properties, as well as α-terpineol acetate (21.25%). There is also *p*-linalool (11.15%), α-pinene (4.41%), and α-terpineol (4.83%), compounds with anti-inflammatory, antimicrobial, and bronchodilatory properties. These findings align with the study by Anwar et al. [28], which highlighted linalool as a principal component at 29.1%, followed by 1,8-cineole at 18.4%. While other studies have found that α-pinene is the major constituent of *M. communis* EO [29,30].

It is noteworthy that some molecules are common among these essential oils. For example, cineole is present in all three oils, with particularly high concentrations in *L. dentata* (37.27%) and *M. communis* (43.32%). Similarly, α-pinene is found in *L. dentata* (6.34%), *R. officinalis* (6.10%), and *M. communis* (4.41%), demonstrating its common antibacterial and antifungal properties. Camphor, known for its antiseptic and stimulating effects, is also a common compound between *L. dentata,* at 6.73%, and *R. officinalis,* at 15.00%.

Overall, these studies underscore the influence of ecological, climatic, and nutritional factors on the quantitative and qualitative composition of EOs in plants, corroborating the significant impact of external and internal plant factors, including climate, seasonal variations, soil composition, and metabolic pathways on their chemical profiles [31,32,33].

**Table 1:** Phytochemical profile of *L. dentata*, *R. officinalis*, and *M. communis* EOs using GC-MS.

Compound *	Composition (%)			Linear Retention Index (RI) [34]		Identification
	LDEO	ROEO	MCEO	RI_Calc_ **	RI_Lit_ ***	
**Propanoic acid, 2-methyl-, propyl ester**	-	-	0.76	895	-	RI, MS
α-Pinene	1.30	6.10	4.41	939	935	RI, MS
**Camphene**	-	**11.03**	-	951	950	RI, MS
β-Pinene	6.34	-	1.50	980	981	RI, MS
β-Myrcene	-	1.83	-	993	991	RI, MS
(+)-4-Carene	-	0.89	-	1011	1010	RI, MS
β-Cymene	-	4.14	2.03	1017	1029	RI, MS
D-Limonene	-	8.00	-	1019	1030	RI, MS
**Cineole**	**37.27**	4.97	**43.32**	1029	1036	RI, MS
γ-Terpinene	-	2.18	-	1040	1039	RI, MS
Linalool oxide	1.35	-	-	1054	1070	RI, MS
Ocimene	-	1.62	-	1068	1048	RI, MS
6-Methyl-2-(2-oxiranyl)-5-hepten-2-ol	2.00	-	-	1072	-	RI, MS
β-Linalool	2.41	-	-	1091	1092	RI, MS
***p*** **-Linalool**	-	6.86	**11.15**	1098	1105	RI, MS
**Pinocarveol**	**12.67**	-	-	1125	1136	RI, MS
β-Pinone	2.80	-	-	1134	-	RI, MS
**Camphor**	6.73	**15.00**	-	1145	1151	RI, MS
Borneol	-	4.02	-	1185	1179	RI, MS
Pinocarvone	4.09	1.20	-	1186	1162	RI, MS
*p*-menth-1-en-8-ol	3.34	-	-	1192	1201	RI, MS
Terpinen-4-ol	-	3.74	-	1195	1193	RI, MS
α-Terpineol	-	3.04	4.83	1229	1201	RI, MS
α-Thujenal	-	-	1.24	1243	1246	RI, MS
Myrtenal	4.96	-	-	1262	1260	RI, MS
Pulegone	3.66	-	1.32	1279	-	RI, MS
*cis*-Myrtanyl acetate	-	-	1.94	1286	-	RI, MS
**Bicyclo [3.1.1]hept-2-ene-2-methanol, 6,6-dimethyl**	**6.89**	-	-	1288	-	RI, MS
L-(-)-Carvone	1.76	-	-	1291	1287	RI, MS
**Verbenone**	-	**16.90**	-	1293	1290	RI, MS
Borneol, acetate	-	2.37	-	1302	1299	RI, MS
**α-Terpineol acetate**	-	-	**21.25**	1352	1350	RI, MS
*trans*-Verbenol	-	2.40	-	1356	1359	RI, MS
Terpinyl acetate	-	-	1.26	1367	1360	RI, MS
Geranyl acetate	-	-	2.73	1379	1382	RI, MS
Eugenol methyl ether	-	-	2.26	1395	1407	RI, MS
Caryophyllene oxide	0.78	-	-	1625	1594	RI, MS
β-Selinenol	1.65	-	-	1680	-	RI, MS
MH	7.64	37.97	7.94			
OM	77.38	60.5	81.79			
SH	2.43	-	-			
OS	-	-	-			
Others	12.55	-	10.27			
**Total**	**100**	**98.47**	**100**			

* Components determined by MS and RI; ** Retention indices derived from capillary column (C8–C24) alkanes series; *** linear retention indices sourced from data libraries (NIST), and ADAMS. Hydrocarbons of monoterpenes (MH), oxygenated monoterpenes (OM), hydrocarbons of sesquiterpenes (SH), and oxygenated sesquiterpenes (OS). Major components in each oil are indicated in bold.

### 2.2. Antioxidant Activity of Individual EOs

**Figure 1:**
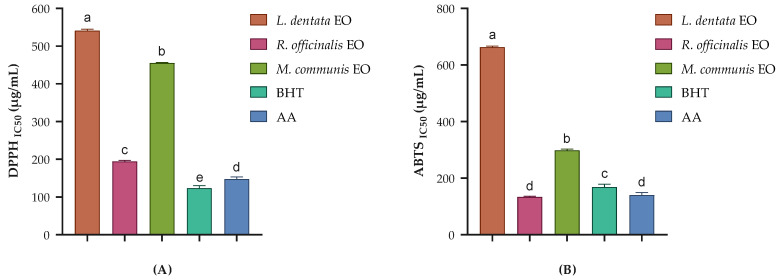
Antioxidant activity of the studied EOs through the DPPH assay (**A**) and ABTS test (**B**). Butylated hydroxytoluene (BHT) and ascorbic acid (AA) were used as standards. Data presented as mean ± SD of three independent experiments. Different letters indicate a statistically significant difference between the groups at *p* < 0.05.

The antioxidant activity of the EOs from *L. dentata*, *R. officinalis*, and *M. communis* was evaluated using two widely accepted methods: the DPPH and ABTS radical scavenging assays (Figure 1). These assays are complementary, as they measure antioxidant capacity through different mechanisms and in different reaction media [35,36]. All three EOs showed higher antioxidant activity in the ABTS assay compared to the DPPH assay. This difference can be explained by the nature of the two assays: ABTS is applicable to both hydrophilic and lipophilic antioxidant systems, while DPPH is more suited for hydrophobic systems [37,38].

*R. officinalis* exhibited the strongest antioxidant activity among the tested EOs, with IC_50_ values of 194.10 ± 3.01 µg/mL in the DPPH assay and 134.07 ± 1.70 µg/mL in the ABTS assay. Interestingly, in the ABTS assay, *R. officinalis* EO exhibited higher antioxidant activity than BHT (IC_50_ = 168.22 ± 10.23 µg/mL) and AA (IC_50_ = 140.22 ± 8.99 µg/mL). This superior activity aligns with previous studies. Moghadam [39] reported high antioxidant activity in *R. officinalis* EO from various regions and cultivars. Interestingly, Beretta et al. [40] observed that the antioxidant capacity of *R. officinalis* EO varies with the plant’s growth stage, peaking at the flowering stage due to the presence of hydroxylated derivatives. Additionally, Pistelli et al. [41] reported that the essential oils from *Rosmarinus officinalis* cultivars showed antioxidant activity, with verbenone and camphor being the main components. The strong antioxidant activity of *R. officinalis* EO can be attributed to its chemical profile, which reveals high concentrations of verbenone (16.90%), camphor (15.00%), camphene (11.03%), and D-limonene (8.00%). These compounds, particularly verbenone and camphor, are known for their antioxidant properties [41]. Verbenone, the most abundant component, has been reported to possess significant free radical scavenging activity [42]. The synergistic effect of these compounds likely contributes to the superior antioxidant activity of *R. officinalis* EO.

*M. communis* EO demonstrated notable antioxidant activity, with IC_50_ values of 455.32 ± 1.21 µg/mL in the DPPH assay and 298.20 ± 4.36 µg/mL in the ABTS assay. This finding aligns with previous studies. Snoussi et al. [43] found that the EO of *M. communis* floral buds exhibited significant antioxidant activity in both β-carotene bleaching and DPPH assays. Similarly, Gardeli et al. [44] reported significant DPPH scavenging activity for *M. communis* EO. These studies support our results and underscore the potential of *M. communis* EO as a natural antioxidant. Its composition is dominated by cineole (43.32%), α-terpineol acetate (21.25%), and *p*-linalool (11.15%). Cineole, the major component, has been reported to exhibit moderate antioxidant activity [45]. The presence of *p*-linalool, known for its antioxidant properties, likely contributes to the overall activity [46]. The relatively high content of oxygenated monoterpenes in *M. communis* EO may explain its stronger performance in the ABTS assay compared to the DPPH assay.

*L. dentata* EO exhibited the lowest antioxidant activity among the three EOs tested, with IC_50_ values of 541.19 ± 3.72 µg/mL in the DPPH assay and 663.42 ± 2.99 µg/mL in the ABTS assay. However, its activity is still noteworthy. Dammak et al. [47] reported moderate antioxidant activity for *L. dentata* EO in DPPH assays, which is consistent with the findings of this study. More recently, Hendel et al. demonstrated significant antioxidant activity for *L. dentata* EO, suggesting that the antioxidant potential of EOs may vary depending on factors such as geographic origin, harvesting time, and extraction method [48,49]. The main components of the studied *L. dentata* EO are cineole (37.27%), pinocarveol (12.67%), camphor (6.73%), and bicyclo[3.1.1]hept-2-ene-2-methanol, 6,6-dimethyl (6.89%). While 1,8-cineole and camphor possess some antioxidant properties, their lower concentrations compared to the other studied EOs may explain the reduced activity.

The variation in antioxidant activity among these three EOs highlights the importance of selecting appropriate EOs for specific antioxidant applications. Moreover, these findings suggest that a combination of these EOs, could potentially yield a synergistic effect, enhancing overall antioxidant activity [10,50].

### 2.3. Simplex Centroid Design

Table 2 details the simplex-centroid design, which includes various mixtures of three essential oils (EO) from *L. dentata*, *R. officinalis*, and *M. communis*, along with their effects (DPPH_IC50_ and ABTS_IC50_) in each test. These oils are known for their health benefits, and studying their combined effects could help create products that reduce oxidative stress and offer other health advantages. Currently, there is no published research on the combined effects of these three oils (*L. dentata*, *R. officinalis*, and *M. communis*) using this method, making this a novel and commendable approach. The study involved 12 randomized trials, and each result is an average of three separate tests [10]. The antioxidant activity measured ranged from 88.67 ± 0.83 to 541.19 ± 3.72 µg/mL for DPPH_IC50_ and 59.33 ± 1.04 to 663.42 ± 2.99 µg/mL for ABTS_IC50_. The analysis indicated that mixture number 11, consisting of *L. dentata*, *R. officinalis*, and *M. communis* in the ratios of 0.17, 0.67, and 0.17, respectively, was the most effective at neutralizing radicals compared to the controls, butylated hydroxytoluene (123.43 ± 6.44 µg/mL for DPPH and 168.22 ± 10.23 µg/mL for ABTS) and ascorbic acid (147.81 ± 5.33 µg/mL for DPPH and 140.22 ± 8.99 µg/mL for ABTS), achieving the lowest IC_50_ values in both the DPPH and ABTS tests.

### 2.4. Statistical Validation of Postulated Model

Table 3 illustrates how variance analysis was utilized to investigate the interactions between the blend’s constituent parts. Since the *p*-values (0.0032 and 0.0110, respectively) were below 0.05, the results showed that the main effects of the regression were statistically significant for both answers (DPPH_IC50_ and ABTS_IC50_). Significant effects were indicated by the estimated F-values for the responses, which were greater than the crucial F-values at the 95% confidence level (17.6865 for DPPH_IC50_, and 10.2207 for ABTS_IC50_).

**Figure 2:**
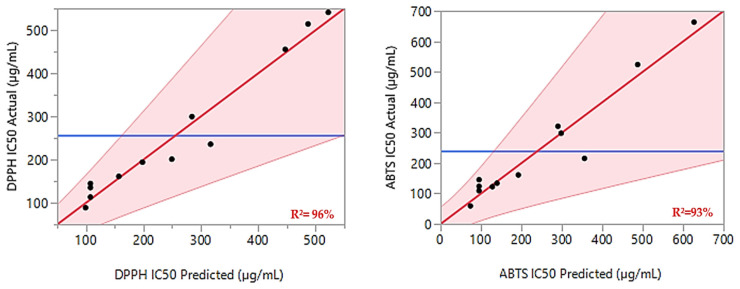
For both responses, DPPH_IC50_ and ABTS_IC50_, the curve of the experimented values in relation to the expected values is shown by the red lines. The actual mean values for the two responses under examination are shown by the blue lines.

Additionally, the ANOVA F-tests confirmed the validity of the models, with *p*-values of 0.0123 and 0.0319, indicating no significant lack of fit. The calculated F-ratios for the lack of fit compared to the pure error were below the critical values (19.16) at a 95% confidence level. High values of the coefficient of determination (R^2^) and adjusted R^2^ (0.96 and 0.90 for DPPH_IC50_, and 0.93 and 0.84 for ABTS_IC50_, respectively) suggest strong agreement between the modeled and observed data. This alignment is further supported by the graph in Figure 2, which displays a linear relationship between the observed and expected values for both responses.

### 2.5. Components Effects and Adjusted Models

The computed regression coefficients for the special model are shown in Table 4. The associations between all tested parameters and the obtained responses for DPPH_IC50_ and ABTS_IC50_ were found using regression models with significant coefficients (*p*-values < 0.05).

The ternary interaction term β123 and the coefficients representing the impacts of the individual components (β1, β2, and β3) are the ones that show the statistical significance for the DPPH_IC50_ response. Nevertheless, the β12, β13, and β23 coefficients of the binary interaction terms have no effect on the DPPH radical and are non-significant (*p* > 0.05). In fact, Equation (1) expresses the mathematical models that describe the response as a function of the tested components after eliminating any non-significant coefficients from the presumed models.

(1) $$Y_{DPPH-IC50}={521.924H}_{1}+{198.364H}_{2}+{446.975H}_{3}-662.160H_{2}H_{3}-4729.370H_{1}H_{2}H_{3}+ɛ$$

Concerning the ABTS_IC50_ response, the significant terms were β_1_, β_3_, and β_123_. These results confirm that the ternary effect and the effects of *M. communis* and *L. dentata* EOs have a major influence on the antioxidant capability against ABTS radicals. Equation (2) thus expresses the accepted mathematical model:

(2) $$Y_{ABTS-IC50}={626.904H}_{1}+{298.164H}_{2}-5106.523H_{1}H_{2}H_{3}+ɛ$$

### 2.6. Desirability and Optimization of the Formulation

The optimization process utilizing the experimental design methodology involves determining the ideal ratios of the components being studied to achieve the best possible response values. Although the optimal results generated by statistically verified mathematical models might not always correspond directly to those observed in the 12 conducted experiments, they are capable of predicting them with considerable accuracy within the experimental scope. To uncover the most favorable responses, testing begins from the highest values obtained. Consequently, the top recorded results were 88.67 ± 0.83 and 59.33 ± 1.04 µg/mL for DPPH_IC50_ and ABTS_IC50_, respectively. The settings that could achieve responses at or above these levels were deemed acceptable.

### 2.7. Mixture Profile

**Figure 3:**
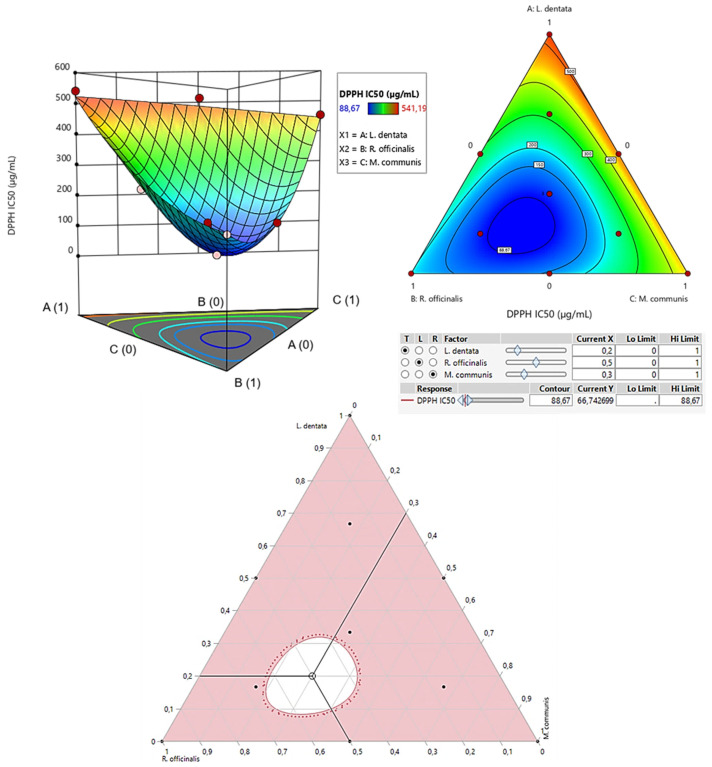
The optimal value of DPPH_IC50_ was obtained by analyzing 2D and 3D mixture plots of the intended compromise area.

The contour plot and 3D surface graph (shown as 2D and 3D mixture plots in Figure 3) display the optimal mix of the three essential oils—*L. dentata*, *R. officinalis*, and *M. communis*—to maximize the responses (DPPH_IC50_ and ABTS_IC50_). These visualizations highlight the relationship between the responses and the concentrations of each antioxidant. Created using Design-Expert software, these plots utilize iso-response curves, ideal for pinpointing the best conditions for achieving optimal response values. In the plots, blue indicates lower IC_50_ values and higher antioxidant effectiveness, while colors transitioning from yellow to dark red represent increasing IC_50_ values, indicating lower effectiveness.

#### 2.7.1. Optimization of DPPH_IC50_

**Figure 4:**
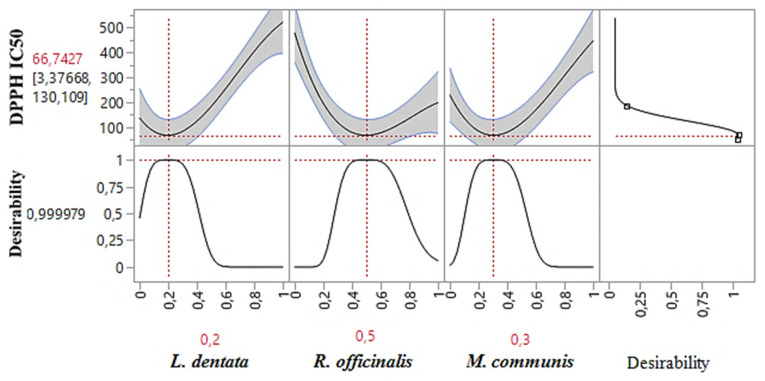
Desirability profile illustrating the precise proportions of *L. dentata*, *R. officinalis*, and *M. communis* EOs, leading to the optimum value for DPPH_IC50_. Red numbers are the best mixture values.

As shown in the 2D and 3D mixture plots (Figure 3), the dark blue area represents the optimal compromise for achieving the best DPPH_IC50_ value, which is 88.67 μg/mL, using a ternary mixture of *L. dentata*, *R. officinalis*, and *M. communis*. The effectiveness of this combination is further confirmed by the desirability test (Figure 4), which indicates that an optimal DPPH_IC50_ value of 66.74 μg/mL, with a desirability of 99%, can be achieved with the following proportions: 20% *L. dentata*, 50% *R. officinalis*, and 30% *M. communis* EOs. This level of antioxidant activity surpasses that of standard antioxidants such as BHT (IC_50_ = 123.43 ± 6.44 μg/mL) and ascorbic acid (IC_50_ = 147.81 ± 5.33 μg/mL).

#### 2.7.2. Optimization of ABTS_IC50_

**Figure 5:**
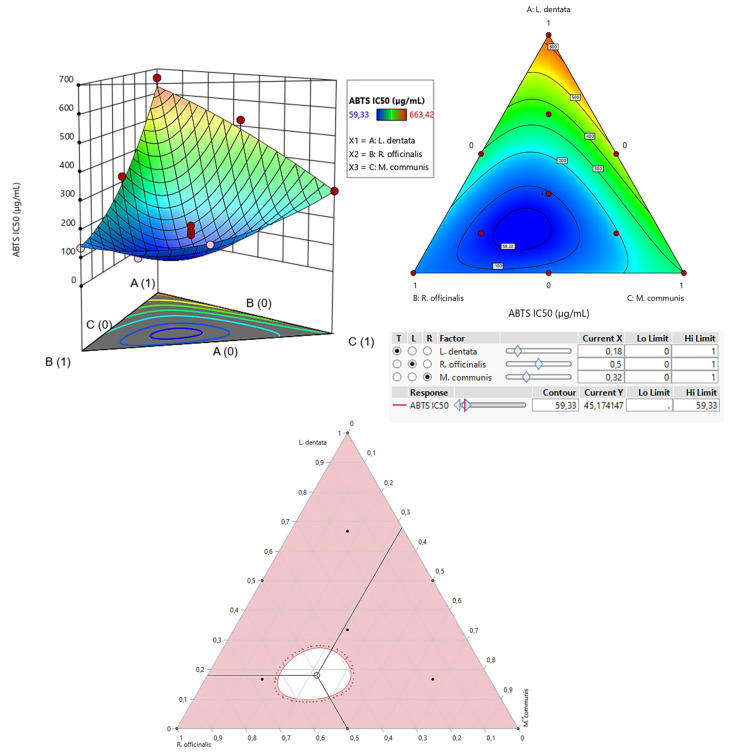
The optimal value of ABTS_IC50_ was obtained by analyzing 2D and 3D mixture plots of the intended compromise area.

**Figure 6:**
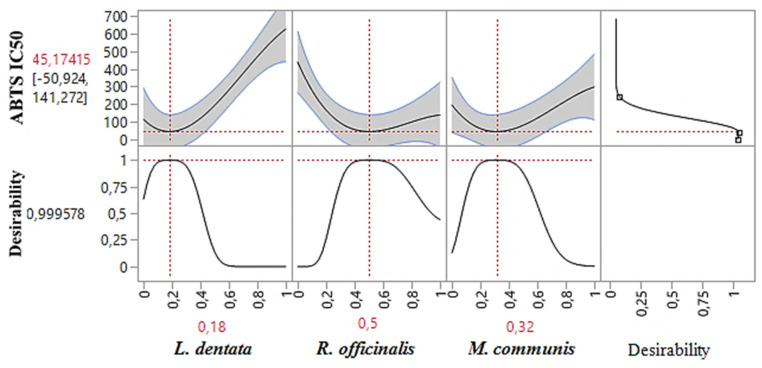
Desirability profile illustrating the precise proportions of *L. dentata*, *R. officinalis*, and *M. communis* EOs, leading to the optimum value for ABTS_IC50_. Red numbers are the best mixture values.

As shown in the 2D and 3D mixture plots (Figure 5), the dark blue area represents the optimal compromise for achieving the best DPPH_IC50_ value, which is 88.67 μg/mL, using a ternary blend of *L. dentata*, *R. officinalis*, and *M. communis*. The effectiveness of this combination is further confirmed by the desirability test (Figure 6), which indicates that an optimal DPPH_IC50_ value of 66.74 μg/mL, with a desirability of 99%, can be achieved with the following proportions: 20% *L. dentata*, 50% *R. officinalis*, and 30% *M. communis* EOs. This level of antioxidant activity surpasses that of standard antioxidants such as BHT (IC_50_ = 123.43 ± 6.44 μg/mL) and ascorbic acid (IC_50_ = 147.81 ± 5.33 μg/mL).

The methodology of mixture design has seen increasing use among researchers in various disciplines, especially in formulating essential oil (EO) mixtures [51]. For example, Baj et al. [52] optimized a blend of EOs from basilic (*Ocimum basilicum* L.), citronella grass (*Cymbopogon nardus* (L.) Rendle), eastern red cedar (*Juniperus virginiana* L.), and thyme (*Thymus vulgaris* L.) to improve DPPH radical scavenging capacity. Similarly, the Simplex Lattice Mixture design was employed to refine the effects of combining EOs from parsley (*Petroselinum crispum* (Mill.)), coriander (*Coriandrum sativum* L.), and celery (*Apium graveolens* L.) [53].

There is increasing interest in exploring the synergistic antimicrobial properties of EO mixtures to enhance their effectiveness. Falleh et al. [54] used this methodology to determine the optimal proportions of EOs from Spanish lavander (*Lavandula stoechas* L.), clove (*Syzygium aromaticum* (L.) Merr. & L.M. Perry), myrtle (*Myrtus communis* L.), and Ceylon cinnamon (*Cinnamomum zeylanicum* Blume). They discovered that a mixture of 59.4% *C. zeylanicum*, 38.2% *L. stoechas*, and 2.4% *S. aromaticum* exhibited synergistic interactions, particularly effective against *Escherichia coli*. Additionally, Assagaf et al. [55] used the same methodology as this study to identify the best proportions of lemongrass (*Cymbopogon fexuosus* (Nees ex Steud.) W. Watson), caraway (*Carum carvi* L.), and sweet flag (*Acorus calamus* L.) for enhanced antioxidant activity. The optimal blend was found to be 20% *C. fexuosus*, 53% *C. carvi*, and 27% *A. calamus* for achieving a lower DPPH_IC50_. Conversely, the most effective combination for the highest scavenging activity against the ABTS radical was determined to be 17% *C. fexuosus*, 43% *C. carvi*, and 40% *A. calamus*.

### 2.8. Simultaneous Optimization of All Responses

**Figure 7:**
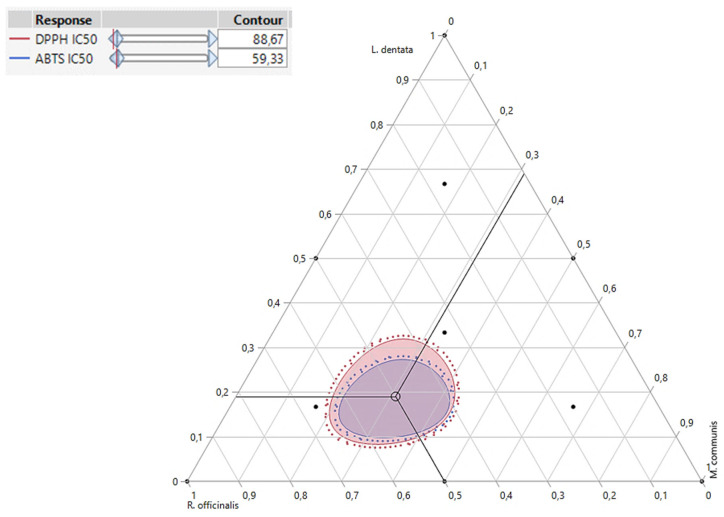
2D and 3D mixture plots of the desired compromise area, resulting in the best value of DPPH_IC50_, and ABTS_IC50_.

**Figure 8:**
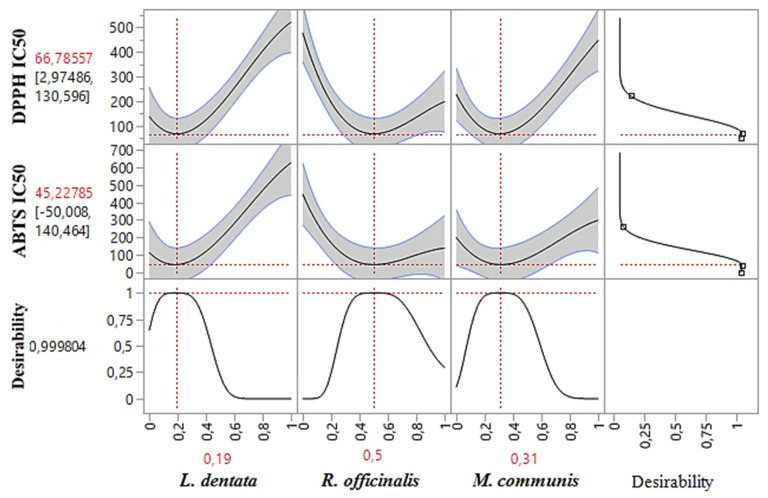
Desirability profile of the simultaneous optimization showing the precise proportions of the three tested EOs, leading to optimal values for the two responses (DPPH_IC50_ and ABTS_IC50_). Red numbers indicate the best mixture values.

Besides accurately predicting DPPH_IC50_, and ABTS_IC50_ responses individually, the desirability test also facilitates the identification of optimal conditions for both responses together. In our study, the goal of simultaneous optimization was to find the most effective compromise for improving both the DPPH_IC50_ and ABTS_IC50_ responses. As shown in the desirability graph (Figure 7 and Figure 8), we achieved this with a near-perfect compromise efficiency of 99.98%, using a ternary blend of 19% *L. dentata*, 50% *R. officinalis*, and 32% *M. communis* EOs. For this specific mixture, the optimal response values were determined to be 66.78 μg/mL for DPPH_IC50_ and 45.22 μg/mL for ABTS_IC50_.

### 2.9. Experimental Verification of the Assumed Model

Table 5 offers a detailed verification of cubic models used to evaluate the antioxidant effects of a combination of essential oils (EOs) from *L. dentata*, *R. officinalis*, and *M. communis*. This analysis is essential for confirming the precision of these models in predicting the antioxidant activities, measured through the DPPH_IC50_ and ABTS_IC50_ assays. The model’s accuracy is substantiated by aligning experimental results with predicted outcomes, showcasing their effective correlation and demonstrating the reliability of the model in practical applications.

In the specific results shown, the mixture consists of 19% *L. dentata*, 50% *R. officinalis*, and 31% *M. communis*. The experimental value for DPPH_IC50_ was recorded at 71.23 ± 0.98 µg/mL, closely matching the predicted value of 66.78 ± 00.00 µg/mL, while the ABTS_IC50_ had an experimental value of 44.39 ± 1.07 µg/mL, aligning well with the predicted value of 45.22 ± 00.00 µg/mL. These results highlight the capability of the model to accurately predict the antioxidant potential of these EO combinations under tested conditions.

The validation of these results is critical as it not only supports the reliability of the modeling approach but also contributes to the broader understanding of how specific proportions of various EOs can synergistically enhance antioxidant effects.

## 3. Materials and Methods

### 3.1. Plant Material and Extraction of EOs

*Lavandula dentata* L. (aerial parts), *Rosmarinus officinalis* L. (leaves), and *Myrtus communis* L. (leaves) were collected from the local experimental station at the Faculty of Sciences, University Mohammed the First, Oujda, in northeastern Morocco (34°39′7.562″ N, 1°54′0.812″ W), voucher numbers for the collected plants are HUMPOM76, HUMPOM84, HUMPOM104, respectively. The plants were identified by Pr. Mohamed ADDI at the same institution. This study adhered to all relevant guidelines and regulations. The samples were dried under continuous ventilation in a dark place, and essential oils (EOs) were extracted by hydro-distillation [56]. Specifically, 100 g of dried plant material was distilled for 180 min using a Clevenger-type apparatus. The resulting oils were collected, dehydrated with anhydrous sodium sulfate, filtered, and stored at 4 °C for further analysis.

### 3.2. Chemicals

Butylated hydroxytoluene (BHT), ascorbic acid (AA) and 1,1-diphenyl-2-picryl-hydrazyl (DPPH) were bought from Sigma-Aldrich Chemical Co. (St. Louis, MO, USA). Further, 2,2′-azino-bis(3-ethylbenzothiazoline-6-sulfonic acid) (ABTS) and dimethylsulfoxide were also brought from Sigma-Aldrich Chemical Co. (St. Louis, MO, USA).; sodium persulfate and ethanol were from Merck (Merck Chemicals, Saint-Quentin Fallavier, France). All other reagents were of analytical grade.

### 3.3. GC-MS Analysis of EOs

The volatile constituents of the three essential oils were analyzed using gas chromatography (GC) connected to a Shimadzu GC/MS-QP2010 series mass spectrometer [57,58]. Samples were vaporized and injected via a split/splitless injector into a BP-X25 capillary column (30 m × 0.25 mm) coated with a non-polar stationary phase (95% dimethylpolysiloxane/5% phenyl). Helium served as the carrier gas at a flow rate of 3 mL/min. The setup included set temperatures for the injection, ion source, and interface at 250 °C. The column oven’s temperature was initially held at 50 °C for 1 min, then ramped to 250 °C at a rate of 10 °C/min for 1 min. Ionization of the sample components occurred using EI mode (70 eV) with a mass scan range from 40 to 300 *m*/*z*. The compounds were separated and detected using a mass spectrometer, and their identities confirmed by matching retention times and mass spectral fragmentation patterns against known standards and databases, including the National Institute of Standards and Technology (NIST) [59] and ADAMS [60]. Data collection and analysis were conducted using LabSolutions software (version 2.5).

### 3.4. Antioxidant Assays

#### 3.4.1. DPPH Radical Scavenging Assay

The antiradical activity of three essential oils (EOs) and their various combinations, generated using an experimental design approach, was assessed using 2,2-diphenyl 1-picrylhydrazyl (DPPH). A modified protocol based on Elbouzidi et al. (2024) [4] was used. Specifically, 800 µL of methanol-prepared samples at different concentrations were mixed with 2 mL of DPPH solution (1 mM). These mixtures were incubated in the dark for 30 min. After incubation, optical density was measured at 517 nm using a spectrophotometer. Butylated hydroxytoluene (BHT) and ascorbic acid (AA) served as references. The radical scavenging activity (RSA) of the EOs was calculated using Equation (3):

(3) $$RSA\left(\%\right)=\left[\left(\frac{A_{b}-A_{x}}{A_{b}}\right)\right]\times 100$$

The IC_50_ value, indicating the concentration required to inhibit 50% of the free radicals, was determined by plotting the inhibition percentage against the extract concentration. This calculation utilized the absorbance values of the blank (*A_b_*) and the sample or positive control (*A_x_*).

#### 3.4.2. ABTS Radical Scavenging Activity

The ABTS radical scavenging method was conducted following the protocols by El Hachlafi et al. [5], with slight modifications. In summary, the ABTS radical cation was generated by mixing 10 mL of ABTS solution (2 mM) with 0.1 mL of potassium persulfate solution (70 mM). This mixture was kept in the dark for 14 h. Subsequently, it was diluted with methanol until an optical density of 0.700 ± 0.02 at 734 nm was achieved. Samples of 0.2 mL at varying concentrations were then combined with 2 mL of the diluted ABTS solution. After 2 min of incubation, the optical density was measured at 734 nm against a methanol blank. The ABTS scavenging activity was expressed as IC_50_ (μg/mL) ± SD (*n* = 3), with butylated hydroxytoluene (BHT) and ascorbic acid used as reference standards.

### 3.5. Experimental Design

#### 3.5.1. Mixture Design

An augmented simplex-centroid design was employed to determine the optimal antioxidant effect of the combined essential oils (EOs) of *L. dentata*, *R. officinalis*, and *M. communis*, as outlined by Benkhaira et al. [61]. The components of the EO system are detailed in Table 6. Each EO in the mixture can range in value from 0 to 1, with the total sum of the three components equaling 1 (Table 1). The antioxidant capacity of the EOs was evaluated using the DPPH_IC50_ and ABTS_IC50_ responses.

#### 3.5.2. Experimental Matrix and Mathematical Model

**Figure 9:**
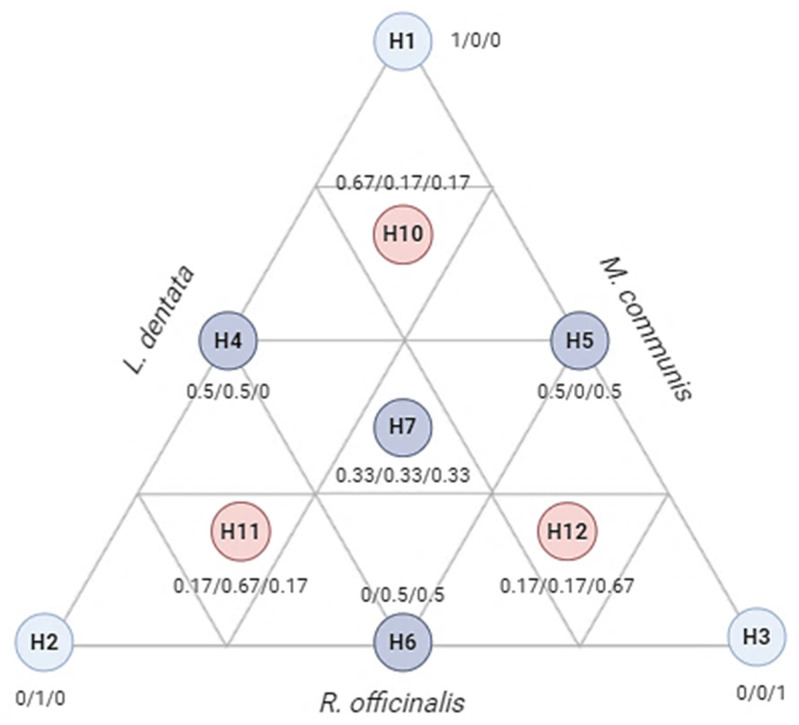
Equilateral triangle of the arrangement of mixtures using the simplex centroid design method. H1: *L. dentata* EO; H2: *R. officinalis* EO; H3: *M. communis* EO.

In this study, a total of 10 trials were conducted and represented as an equilateral triangle (Figure 9). The triangle includes three pure components (1) corresponding to the triangle’s apexes (H1, H2, H3), binary combinations (0.5/0.5) marked by the midpoints of the triangle (H4, H5, H6), and an equal proportion of the three components (0.33/0.33/0.33) located at the triangle’s centroid (H7). This trial was repeated three times, with three control points (H10, H11, H12) representing ternary mixtures (0.67/0.16/0.16). A cubic model was used to express the responses based on the independent variables, as shown in Equation (4):

(4) $$Y={\beta_{1}H}_{1}+{\beta_{2}H}_{2}+{\beta_{3}H}_{3}+\beta_{12}H_{1}H_{2}+\beta_{13}H_{1}H_{3}+\beta_{23}H_{2}H_{3}+\beta_{123}H_{1}H_{2}H_{3}+ɛ$$

where Y is the experimental response determined by IC_50_ (µg/mL); β_1_, β_2_, and β_3_ are linear regression coefficients, β_12_, β_13_, and β_23_ are binary regression coefficients, β_123_ is the ternary regression coefficient, and ɛ is the regression error term.

### 3.6. Statistical Analysis and Optimisation Tools

The Shapiro–Wilk test was used to confirm normality and the Levene’s test for homogeneity of variances. The statistical significance of the mathematical model was assessed at a 95% confidence level using the F-ratio mean square regression/mean square residual (MSR/MSr), which compares the mean square regression to the mean square residual; higher F-values indicate greater variability in the results [62]. Additionally, the ratio of the mean square lack of fit to the mean square pure error (MSLOF/MSPE) was analyzed to evaluate the model’s adequacy, with higher values suggesting potential inadequacies. The coefficient of determination (R²) was calculated to assess model quality. The significance of estimated factors was evaluated using the Student’s t-test, while ANOVA’s F-test confirmed the overall model significance. Analyses were conducted using Design Expert software version 12 and SAS JMP^®^ version 14, with results presented as means ± SD (*n* = 3).

For optimization, contour plots and 3D surface plots illustrated trade-off areas among the studied components. The desirability tool was employed to identify the optimal values, balancing factors for the best outcome. This tool adjusts the mathematical model within a range of 0 to 1, where 0 indicates an undesirable response and 1 signifies a highly desirable response.

## 4. Conclusions

The demand for natural formulations continues to grow. Essential oils, with their diverse chemical compositions influenced by various factors, have the potential for complex interactions. Utilizing statistical tools, such as experimental mixture design, can effectively optimize these interactions for enhanced biological effects in the food and pharmaceutical industries. Our findings suggest the optimal antioxidant formulation consists of 20% *L. dentata*, 50% *R. officinalis*, and 30% *M. communis*, which demonstrated significant DPPH radical scavenging activity with a IC_50_ of 71.23 ± 0.98 µg/mL. Moreover, a blend of 18% *L. dentata*, 50% *R. officinalis*, and 32% *M. communis* yielded the highest ABTS radical scavenging activity with a IC_50_ of 44.39 ± 1.07 µg/mL, attributing this efficacy to their bioactive compounds. Further cell-based tests are needed, along with other investigations into the pharmacokinetics, pharmacodynamics, and toxicological profiles of these mixtures, to verify their safety and efficacy.

## Figures and Tables

**Table 2:** Matrix of simplex centroid design and results for DPPH_IC50_, and ABTS_IC50_.

No. ^a^	*L. dentata*	*R. officinalis*	*M. communis*	Observed Responses ^b^ (µg/mL)	
				DPPH_IC50_	ABTS_IC50_
1	1	0	0	541.19 ± 3.72	663.42 ± 2.99
2	0	1	0	194.10 ± 3.01	134.07 ± 1.70
3	0	0	1	455.32 ± 1.21	298.20 ± 4.36
4	0.5	0.5	0	299.57 ± 2.17	321.30 ± 1.81
5	0.5	0	0.5	514.22 ± 7.09	523.96 ± 7.44
6	0	0.5	0.5	161.21 ± 1.18	122.64 ± 3.01
7	0.33	0.33	0.33	135.34 ± 4.07	109.21 ± 2.19
8	0.33	0.33	0.33	144.62 ± 2.81	123.88 ± 0.95
9	0.33	0.33	0.33	113.25 ± 1.97	145.51 ± 2.66
10	0.67	0.17	0.17	235.76 ± 3.52	215.42 ± 2.33
11	0.17	0.67	0.17	88.67 ± 0.83	59.33 ± 1.04
12	0.17	0.17	0.67	201.06 ± 0.37	161.22 ± 0.39
BHT *	-	-	-	123.43 ± 6.44	168.22 ± 10.23
AA **	-	-	-	147.81 ± 5.33	140.22 ± 8.99

^a^ After randomization, the experiments were carried out. ^b^ Tests were run in three separate replicas, and the results were reported as means ± SD. * Ascorbic acid (AA); ** butylated hydroxytoluene (BHT).

**Table 3:** Variance analysis for the three fitted models.

DPPH_IC50_	Model	DF	SS	MS	F	*p*-Value
	R	6	268,557.56	44,759.6	17.6865	0.0032 *
	r	5	12,653.57	2530.7		
	Total	11	281,211.13			
	R²	0.96				
	R²_Adjusted_	0.90				
ABTS_IC50_	Model	DF	SS	MS	F	*p*-value
	R	6	345,693.50	57,615.6	10.2207	0.0110 *
	r	5	28,185.81	5637.2		
	Total	11	373,879.31			
	R²	0.93				
	R²_Adjusted_	0.84				

DF: degree of freedom; SS: sum of squares; MS: mean square; R: regression; r: residual; R²: coefficient of determination. * Statistically signifcant at *p* < 0.05.

**Table 4:** Coefficients of the two presumed models and their level of signifcance (*p*-value). * Statistically signifcant at *p* < 0.05.

Term	Coefficients	DPPH_IC50_		ABTS_IC50_	
		Estimation	*p*-Value	Estimation	*p*-Value
*L. dentata* (Mixture)	β_1_	521.924	0.0001 *	626.904	0.0003 *
*R. officinalis* (Mixture)	β_2_	198.364	0.0095 *	139.591	0.1122
*M. communis* (Mixture)	β_3_	446.975	0.0003 *	298.164	0.0092 *
L. dentata * R. officinalis	β_12_	−302.302	0.2715	−371.768	0.3553
L. dentata * M. communis	β_13_	8.639	0.9732	99.498	0.7961
R. officinalis * M. communis	β_23_	−662.160	0.0425 *	−363.007	0.3658
L. dentata * R. officinalis * M. communis	β_123_	−4729.370	0.0163 *	−5106.523	0.0500 *

Bold indicated the statistically significant *p*-value values.

**Table 5:** Expected and observed responses for the test point that the best-fit mixes were able to achieve.

Mixture of Combination	Mixture (%)	DPPH_IC50_ (µg/mL)		ABTS_IC50_ (µg/mL)	
		Exp. ^a^	Predi. ^b^	Exp.	Predi.
*L. dentata*	19%	71.23 ± 0.98	66.78 ± 00.00	44.39 ± 1.07	45.22 ± 00.00
*R. officinalis*	50%				
*M. communis*	31%				

^a^ Exp: The mean of three replicates plus standard error represents the experimental value. ^b^ Predi: The response ± SD, which was determined by the model, is included with the expected value.

**Table 6:** The independent variables within the mixture.

Components	Coded Variables	Level−	Level+
*L. dentata*	H1	0	1
*R. officinalis*	H2	0	1
*M. communis*	H3	0	1
Sum of proportions		1	

## Data Availability

Data are available in the present paper in the Supplementary Materials.

## Supplementary Materials

The following supporting information can be downloaded at: https://www.mdpi.com/article/10.3390/ph17081071/s1, Figure S1. TIC chromatogram of the volatile composition of *L. dentata* EO using GC-MS. Numbers indicate compounds names as in Table S1; Table S1. Compounds found in *L. dentata* EO; Figure S2. TIC chromatogram of the volatile composition of *R. officinalis* EO using GC-MS. Numbers indicate compounds names as in Table S2; Table S2. Compounds found in *R. officinalis* EO; Figure S3. TIC chromatogram of the volatile composition of *M. communis* EO using GC-MS. Numbers indicate compounds names as in Table S3; Table S3. Compounds found in *M. communis* EO.
