# Supplementary material for: Formulation of a Three-Component Essential Oil Mixture from Lavandula dentata, Rosmarinus officinalis, and Myrtus communis for Improved Antioxidant Activity
Source: Pharmaceuticals (Basel). 2024 Aug 15;17(8):1071. doi: 10.3390/ph17081071 (PMC11357427; doi:10.3390/ph17081071)
Supplement: Supplementary file 1 [file pharmaceuticals-17-01071-s001.zip › pharmaceuticals-3138275-supplementary.pdf]

Article

Formulation of a Three-Component Essential Oil Mixture from *Lavandula dentata, Rosmarinus officinalis,* and *Myrtus communis* for Improved Antioxidant Activity

1. Toothed Lavender (*Lavandula dentata* L.)

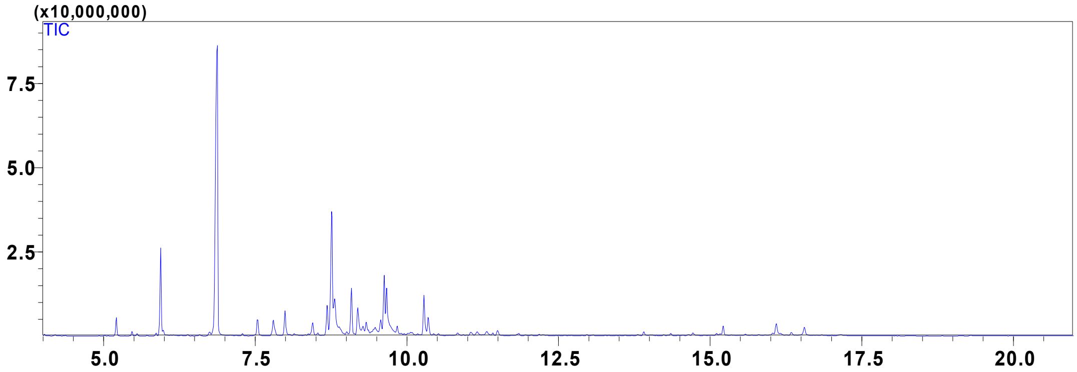

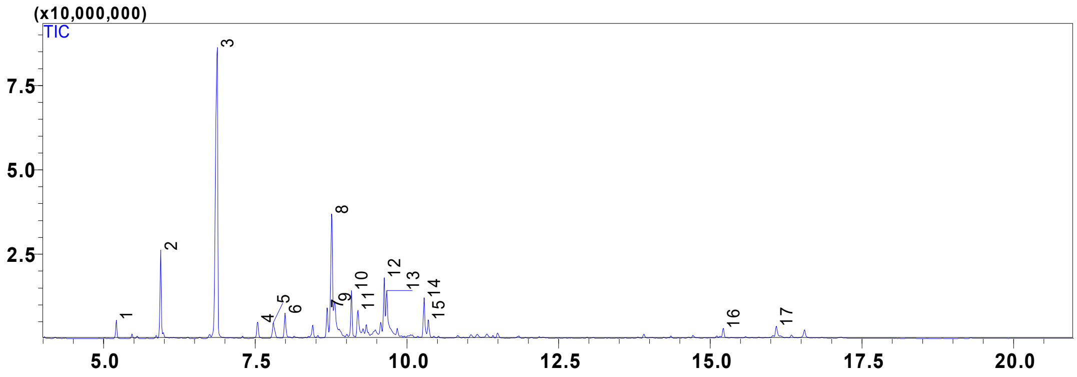

Figure S1. TIC chromatogram of the volatile composition of *L. dentata* EO using GC-MS. Numbers indicate compounds names as in Table S1.

Table S1. Compounds found in *L. dentata* EO.

N	Compounds	R.T (min)	Area (%)
1	α-Pinene	5.207	1.30
2	β-pinene	5.940	6.34
3	Cineole	6.871	37.27
4	Linalool oxide	7.537	1.35

5	6-Methyl-2-(2-oxiranyl)-5-hepten-2-ol	7.798	2.00
6	β-Linalool	7.992	2.41
7	β-Pinone	8.684	2.80
8	Pinocarveol	8.761	12.67
9	Camphor	8.809	6.73
10	Pinocarvone	9.084	4.09
11	*p*-Menth-1-en-8-ol	9.190	3.34
12	Myrtenal	9.625	4.96
13	Bicyclo[3.1.1]hept-2-ene-2-methanol, 6,6-dimethyl	9.666	6.89
14	Pulegone	10.283	3.66
15	Carvone	10.353	1.76
16	Caryophyllene oxide	15.213	0.78
17	β-Selinenol	16.088	1.65

2. Rosemary (*Rosmarinus officinalis* L.)

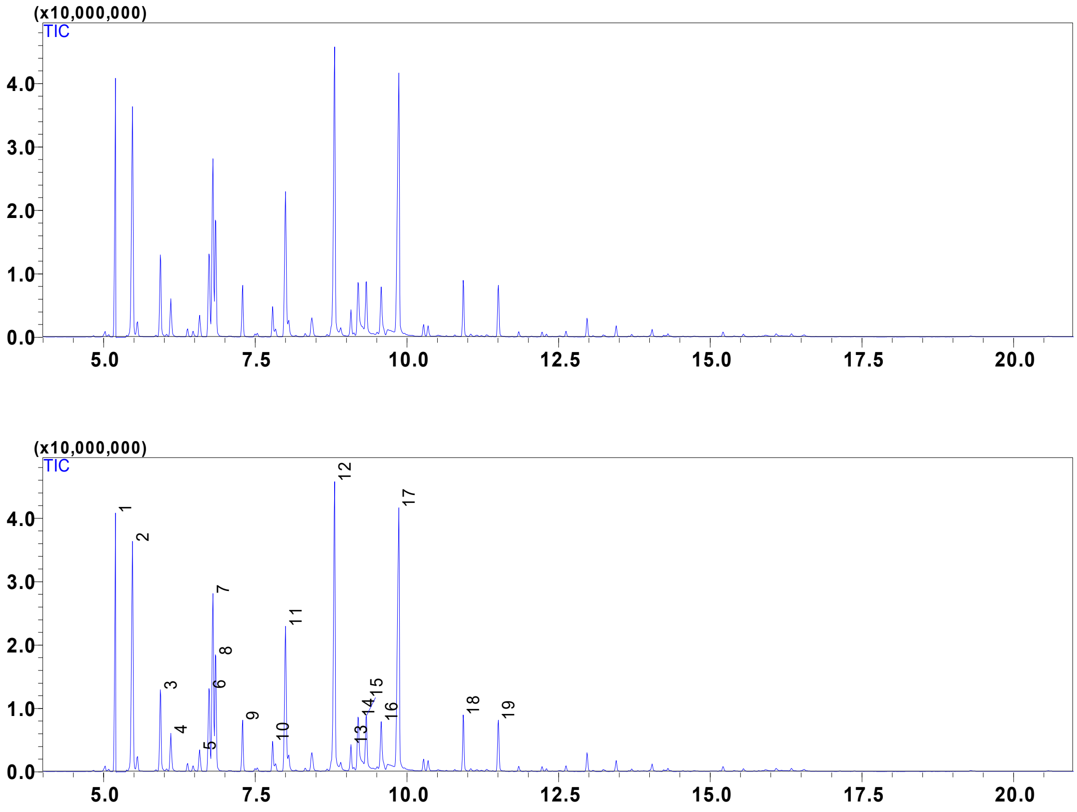

Figure S2. TIC chromatogram of the volatile composition of *R. officinalis* EO using GC-MS. Numbers indicate compounds names as in Table S2.

Table S2. Compounds found in *R. officinalis* EO.

No.	Compounds	R.T (min)	Area (%)
1	α-Pinene	5.190	6.10
2	Camphene	5.475	11.03
3	β-Pinene	5.936	3.71
4	β-Myrcene	6.107	1.83
5	(+)-4-Carene	6.582	0.89
6	β-Cymene	6.736	4.14
7	D-Limonene	6.799	8.00
8	1,8-Cineole	6.846	4.97
9	γ-Terpinen	7.289	2.18
10	Ocimene	7.785	1.62
11	*p*-Linalool	7.999	6.86
12	Camphor	8.806	15.00
13	Pinocarvone	9.074	1.20
14	Borneol	9.195	4.02
15	Terpinen-4-ol	9.329	3.74
16	α-Terpineol	9.574	3.04
17	Verbenone	9.866	16.90
18	Borneol, acetate	10.927	2.37
19	*trans*-Verbenol	11.506	2.40

3. Myrtle (*Myrtus communis* L.)

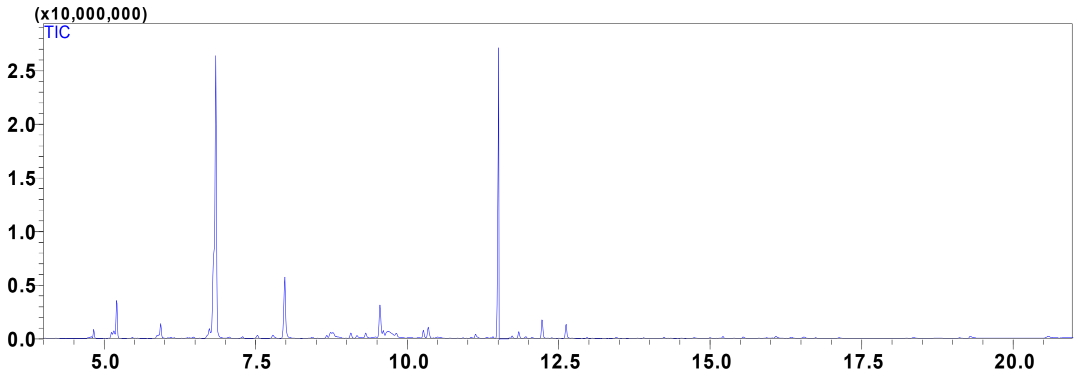

Figure S3. TIC chromatogram of the volatile composition of *M. communis* EO using GC-MS. Numbers indicate compounds names as in Table S3.

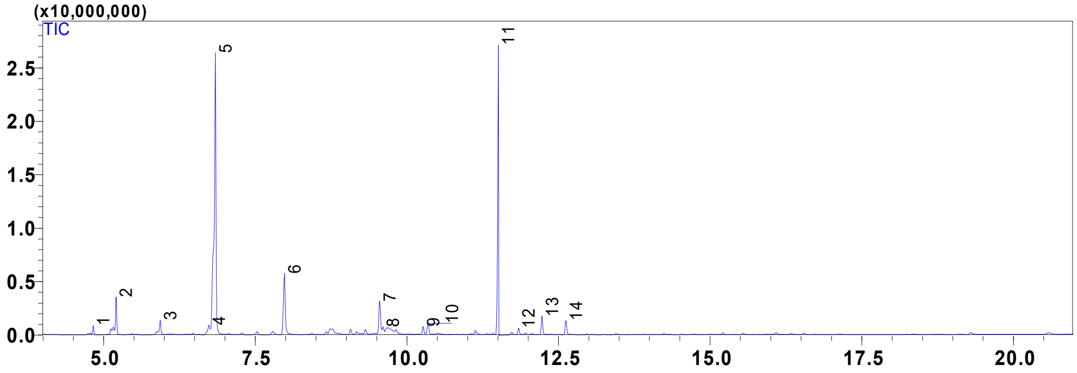

Figure S3. *Continued.*

Table S3. Compounds found in *M. communis* EO.

No.	Compounds	R.T (min)	Area (%)
1	Propanoic acid, 2-methyl-, propyl ester	4.826	0.76
2	α-Pinene	5.203	4.41
3	β-Pinene	5.932	1.50
4	β-Cymene	6.733	2.03
5	1,8-Cineole	6.841	43.32
6	*p*-Linalool	7.981	11.15
7	α-Terpieol	9.550	4.83
8	α-Thujenal	9.607	1.24
9	Pulegone	10.268	1.32
10	*cis*-Myrtanyl acetate	10.351	1.94
11	α-Terpineol acetate	11.506	21.25
12	Terpinyl acetate	11.841	1.26
13	Geranyl acetate	12.229	2.73
14	Eugenol methyl ether	12.623	2.26
